# Risk assessment in equine anesthesia: a first evaluation of the usability, utility and predictivity of the two-part CHARIOT

**DOI:** 10.3389/fvets.2024.1384525

**Published:** 2024-05-23

**Authors:** Lisa Brumund, Liza Wittenberg-Voges, Karl Rohn, Sabine B. R. Kästner

**Affiliations:** ^1^Clinic for Horses, University of Veterinary Medicine Hannover, Foundation, Hannover, Germany; ^2^Institute for Biometry, Epidemiology and Information Processing, University of Veterinary Medicine Hannover, Foundation, Hannover, Germany; ^3^Clinic for Small Animals, University of Veterinary Medicine Hannover, Foundation, Hannover, Germany

**Keywords:** horse, equine, anesthesia, mortality, morbidity, risk assessment, CHARIOT

## Abstract

**Introduction:**

An accurate risk score that can predict peri-anesthetic morbidity and mortality in equine patients could improve peri-operative management, outcome and client communication.

**Materials and methods:**

Three hunded horses underwent pre-anesthetic risk assessment using the American Society of Anesthesiologists-Physical Status augmented with equine-specific diseases (ASA-PS-Equine), a multifactorial 10-part rubric risk scale (10-RS), and a combination of both, the Combined horse anesthetic risk identification and optimization tool (CHARIOT). Intra-and post-anesthetic complications, the recovery phase and mortality were recorded over a period of 7 days following general anesthesia. To compare the utility and predictive power of the 3 scores, data were analyzed using binominal logistic regression (*p* ≤ 0.05) and receiver operating characteristic curve analysis. In addition, inter-observer reliability, speed, safety, ease of use and face validity of the ASA-PS-Equine and the 10-RS were analyzed based on five hypothetical patients.

**Results:**

All scores showed statistically significant associations with various intra-anesthetic complications and parameters of the recovery phase. The discriminant ability of the scores related to the occurrence of intra-anesthetic (AUC = 0.6093–0.6701) and post-anesthetic (AUC = 0.5373–0.6194) complications was only low. The highest diagnostic accuracy for all scores was observed for overall mortality (AUC = 0.7526–0.7970), with the ASA-PS-Equine differentiating most precisely (AUC = 0.7970; 95% CI 0.7199–0.8741). Inter-observer reliability was fair for the 10-RS (κ = 0.39) and moderate for the ASA-PS-Equine (κ = 0.52). Patient assignment to the CHARIOT was predominantly rated as rather easy and quick or very quick.

**Limitations and conclusion:**

The main limitations of the study are the monocentric study design and failure to obtain the full range of points. In conclusion, all 3 scores provide useful information for predicting the mortality risk of equine patients undergoing general anesthesia, whereas intra-and postoperative complications cannot be predicted with these scores.

## 1 Introduction

Most studies on general anesthesia in horses report mortality rates of approximately 1% (1–6). Although several patient- and procedure-specific risk factors for increased equine anesthetic risk have been identified, there is still no validated and accurate risk assessment tool available. The evolving complexity of equine surgical and imaging procedures associated with longer anesthetic durations require improvement in peri-anesthetic management (7). Although originally developed for other purposes (8) and often criticized for its subjectivity (9–16) the ASA Physical Status Classification System (ASA-PS) still serves as the standard method for pre-operative assessment of anesthetic risk in human and veterinary medicine (7, 17). To optimize the pre-anesthetic risk assessment in horses, Hubbell et al. (7) proposed a multifactorial risk index, but its performance has not yet been evaluated. This new risk index incorporates a combination of a modified version of the ASA classification (ASA-PS-Equine) and a 10-part rubric risk scale (10-RS) and is referred to by the authors as the Combined Horse Anesthetic Risk Identification and Optimization Tool (CHARIOT). While the ASA-PS-Equine contains common equine diseases, the 10-RS includes 6 patient-specific risk factors such as age, bodyweight, tractability, mobility, abdominal profile, and pain level, as well as 3 procedure-specific factors like the procedure performed, patient positioning and anticipated duration of anesthesia and the anesthesia-specific assistance during recovery phase. Depending on the distribution of the horses in the categories of each variable, an estimated risk is allocated (1: baseline risk; 2: 2-fold risk; 3: 3-fold risk). A functioning risk assessment tool could facilitate accurate determination of a patient’s anesthetic risk, the required care and, consequently, provide accurate information to the owner regarding the chances of survival and potential costs associated with their animal’s surgical care. Therefore, the aim of the study was to evaluate the usability, utility and predictive power of the CHARIOT in a prospective study involving clinical patients. We hypothesized that the summary CHARIOT improves risk prediction compared to its single components the ASA-PS-Equine or 10-RS.

## 2 Materials and methods

The study was designed as a prospective observational study of horses presented for elective or emergency anesthesia episodes at the Clinic for Horses of the University of Veterinary Medicine Hannover. After each horse was assessed with the ASA-PS-Equine and 10-RS, the CHARIOT was calculated. As this study was purely observational and no horse would receive specific treatment as a result of the study, approval from the institutional ethical committee of the University of Veterinary Medicine Hannover was waived prior to the start of data collection. Written consent on data use was obtained from all owners.

### 2.1 Animals

The Study included 300 horses that underwent general anesthesia for surgical or non-surgical procedures between February 2022 and December 2022. Exclusion criteria comprised: failure to take a photograph before general anesthesia and missing documentation of a patient’s physical status, course of anesthesia, parameters of the recovery phase or post-anesthetic period. Due to the absence of studies on this particular scoring system, no *a priori* sample size calculation was performed. Therefore, a required sample size of 300 participants was estimated based on average values from the literature with documented anesthesia-associated mortality rates of 1% in equine patients (1–6).

### 2.2 ASA-PS-equine classification

To classify the horses into the ASA-PS-Equine categories proposed by Hubbell et al. (7), the digital patient record (easyVET^®^, VetZ GmbH, Germany) was examined for existing diseases of the patients. According to pre-existing conditions, horses were assigned to ASA-PS-Equine grade 1 to 5. If a horse comprised conditions that were not listed by Hubbell et al. (7), the assignment was based on the suspected systemic effects on the physical status caused by the current condition in consultation with the authors (LB, LW, SK).

### 2.3 10-RS classification

For each of the 10 categories of the 10-RS, namely age, bodyweight, tractability, mobility, abdominal profile, pain level, procedure, recumbency, anticipated anesthetic duration, and assistance during recovery phase a risk score value between 1 and 2 or 1 and 3 was assigned (7). For the categories age, bodyweight, mobility, procedure, recumbency, and planned assistance during the recovery phase, the information for each patient was retrieved from the patient record. If surgical procedures were performed that were not included in the original description of the 10-RS (7), risk assessment of these procedures was performed pre-operatively in agreement by the authors (LB, W, SK) as follows:

1. Minor orthopedic surgery (arthroscopy, bursoscopy, tendovaginoscopy, desmotomy, neurectomy, hoof procedures), diagnostic imaging (CT, MRI), urogenital surgery (castration, cryptorchidectomy, penile reposition), laceration.
2. Head surgery (bone flap, tooth extraction, osteosynthesis mandibular fracture, keratohyoidectomy, subepiglottic cyst resection, ophthalmologic procedures), vascular surgery (surgical hemostasis, trans-arterial coil embolization, trans-venous electrical cardioversion, phlebectomy), laparotomy, omphalectomy, phallectomy, myelography, splint bone surgery (distal fragment).
3. Osteosynthesis (including removal of proximal splint bone fragments), arthrodesis.

A short summarizing assessment of the tractability of the equine patients was performed by the attending veterinarian during physical examination, placement of an intravenous catheter and while leading the horses into the examination room. According to this, horses were divided into easy or difficult to handle (7). Horses with signs of pain that could be suppressed by the administration of analgesic drugs or sedatives were classified as “controllable painful.” Horses that did not show significant improvement of clinical signs of pain (e.g., lameness, colic symptoms) despite pain medication were assigned to the “uncontrollable painful” category. Prior to general anesthesia, a side view photograph was taken of each horse. Using an image processing program (ImageJ, U.S. National Institute of Health, United States), the abdominal profile of the patients was examined depending on the outline of the ventral abdomen [based on (18)]. To determine the anticipated anesthesia time, the duration of different clinic-specific procedures was estimated by 3 specialized anesthetists, 3 surgeons and 3 veterinary technicians. The majority of the ratings for the estimated anesthesia times were determined as reference value and additionally compared with the effective anesthesia duration. After the horses were given a score for the ASA-PS-Equine (points 1–5) and 10-RS (points 10–23), both scores were added up to obtain the CHARIOT (points 11–28).

### 2.4 Anesthetic management and monitoring

For elective procedures food but not water was withheld for 6 h prior to anesthesia, foals and horses undergoing emergency surgery did not fast. Anesthetic protocol and ventilation strategy were determined by the anesthetist. Following the induction of anesthesia and endotracheal intubation, horses were positioned depending on the procedure performed and supplied with 100% oxygen, for total intravenous anesthesia (TIVA, triple drip: ketamine, xylazine, guaifenesin) or inhalational anesthesia (isoflurane). In the majority of cases, an arterial catheter was placed (transverse facial, facial or metatarsal artery) to permit invasive arterial blood pressure measurement and blood gas analysis. Via fluid-filled low compliance extension lines the arterial catheter was connected to a pressure transducer (Meritrans DTXPlus^®^, Merit Medical Ireland Ltd., Ireland), which was positioned at the level of the heart and zeroed to atmospheric pressure. When anesthetic duration was less than 20 min, blood pressure was measured noninvasively at the coccygeal artery by high definition oscillometry (VET HDO MD Equine, S + B medVET GmbH, Germany). Intermitted measurement of arterial blood gasses and electrolytes were performed with a standard blood gas analyzer (ABL800 FLEX, Radiometer GmbH, Germany). Peripheral oxygen saturation (SpO_2_) was measured by a portable pulse oximeter (LiveVet^®^PT, Eickemeyer Medizintechnik für Tierärzte KG, Germany). Electrocardiography, capnography and gas analysis was performed using a multiparameter monitor (Carescape B450/Datex-Ohmeda, GE-Healthcare Finland Oy, Finland). Body temperature of the horses was measured with an intra-nasal temperature probe or rectally with a standard digital thermometer. Heart rate, mean arterial pressure (MAP), SpO_2_ and end tidal carbon dioxide concentration (EtCO_2_) were measured continuously and recorded in an anesthesia protocol at 10-minute intervals. Any incident occurring during anesthesia was recorded at the time of the occurrence.

### 2.5 Intra-anesthetic complications

Occurrence of intra-anesthetic complications, namely hypotension, hypoxemia, hypothermia, hypercarbia, cardiac arrhythmias, bleeding, excitation, intra-anesthetic awakening, death/euthanasia was extrapolated from the anesthetic records. Intra-anesthetic complications were defined as follows:

- Hypotension: MAP <70 mmHg for at least 15 consecutive minutes (19).
- Hypoxemia: PaO_2_ < 60 mmHg or SaO_2_/SpO_2_ < 90% for at least 15 consecutive minutes (19).
- Hypothermia: body temperature < 35°C for at least 15 consecutive minutes (19).
- Hypercarbia: PaCO_2_ or EtCO_2_ > 65 mmHg for at least 15 consecutive minutes (19).
- Cardiac arrhythmias: any disturbance of the cardiac rhythm that can be detected with electrocardiography, among bradycardia (heart rate < 20 beats/min), atrial fibrillation, ventricular fibrillation, ventricular fluttering (20, 21).
- Bleeding: blood loss requiring treatment (e.g., volume replacement, surgical ligature) (19).
- Excitation: mild state of arousal with muscle movements during premedication, induction or recovery phase.
- Intra-anesthetic awakening: rapid nystagmus or movement requiring additional anesthetic drugs (19).

Anesthesia-associated mortality was defined as mortality associated with a procedure performed under general anesthesia if anesthesia was associated with the death of a horse but was not solely responsible because patient- or procedure-specific risk factors contributed to it (22). All other deaths were categorized as euthanasia.

### 2.6 Recovery management

After general anesthesia, horses were transferred to a padded recovery box. Intra-nasal oxygen supplementation was provided with a flow rate of 10 L/min, until movement of the horses dislodged the oxygen line. Most adult horses were sedated with xylazine (0.1–0.2 mg/kg IV, CP-Pharma, Germany) as soon as nystagmus was detected. Once the swallow reflex in adult horses returned the endotracheal tube (Cuffed Endotracheal Tube, ET 22–30 mm, Smiths Medical PM, United States) was replaced by a silicone nasopharyngeal tube (Equine Post Anesthetic Nasal Tube, ENAT14, Smiths Medical PM, USA). Assistance during recovery was always provided with head-tail-ropes (23), occasionally accompanied with sling systems (TBTN^®^, Ruedi Keller, Switzerland; Equi-Lift-Hebegeschirr, MedVet, Germany) in adult horses. Foals or small ponies were supported manually. For each recovery phase, duration (time from turning vaporizer off/stop infusion of anesthetic drugs to standing) and attempts to stand were recorded. Recovery quality was scored by the attending anesthetist by using a modified scale of Young and Taylor (24).

### 2.7 Post-anesthetic monitoring

Following general anesthesia, patients were monitored over a seven-day period for post-anesthetic complications including fractures, neuropathy, myopathy, post-anesthetic colic, epistaxis, respiratory disease, thrombophlebitis, and spontaneous death or euthanasia. Information regarding these complications were taken from the patient records.

### 2.8 Validity and reliability

Information about the health status, planned surgery and a side-view photograph of five hypothetical patients was provided to 12 veterinarians with at least 1 year of experience in equine anesthesia by a questionnaire. Participants were asked to score the ASA-PS-Equine and 10-RS for all patients. Four additional questions were designed to evaluate the usability of the CHARIOT in terms of time investment, ease of performing the score, confidence in correct assignment and to estimate face validity. Participants were asked how quickly they were able to score the patients (very quickly, quickly, within a reasonable time, slowly, or very slowly) and how difficult the CHARIOT was to use (easy, rather easy, not easy/not difficult, rather difficult, or difficult). Additionally, perceived confidence in use was rated (sure, rather sure, not sure/unsure, rather unsure, unsure). Face validity of the CHARIOT was evaluated asking the participants if they consider the tool useful in everyday clinical practice. For each scale, the inter-observer agreement was assessed by comparing the given scores for the ASA-PS-Equine and 10-RS.

### 2.9 Data analysis

Data was transferred to Excel Spreadsheets (Excel, Microsoft Corporation, United States) and analyzed using SAS^®^ statistical software, version 9.4 M7 with SAS Enterprise Guide, version 7.15 (SAS Institute Inc., United States) and GraphPad Prism version 9 for windows (GraphPad Software Inc., United States). Standard basic descriptive statistics were performed, and the model residuals of interval-scaled data (weight, age, duration of anesthesia and recovery) were tested for normal distribution using the Kolmogorov–Smirnov test and visual assessment of the q-q plots. Depending on the distribution, results are given as mean ± standard deviation or median with minimum and maximum. *p*-values ≤0.05 were considered statistically significant. Binominal logistic regression with calculation of the odds ratio were used to assess the association between the ASA-PS-Equine, 10-RS and CHARIOT and the occurrence of intra-and post-anesthetic complications (dichotomous target variables). By Spearman’s rank correlation, it was evaluated whether there is a correlation between the ASA-PS-Equine, 10-RS and CHARIOT and the duration of recovery. Independence between the ASA-PS-Equine, 10-RS and CHARIOT and the attempts to stand and quality of recovery was tested using Fischer’s exact test via Monte Carlo simulation. By receiver operating characteristics curve analysis with calculation of the area under the curve (AUC), the significance of the 3 scores on the occurrence of intra-and post-anesthetic complications and on the mortality rate was determined and compared. Via Fleiss Kappa (κ) coefficients, the inter-observer reliability of the ASA-PS-Equine and the 10-RS was assessed based on the scores given by the participants for hypothetical patients. Based on Landis and Koch (25) κ coefficients were interpreted, with a κ of <0 indicating poor, 0.01–0.2 slight, 0.21–0.4 fair, 0.41–0.6 moderate, 0.61–0.8 substantial, and 0.81–0.99 almost perfect agreement.

## 3 Results

### 3.1 Summary and distribution of subjects

The study population of 300 horses included 139 mares, 126 geldings, and 35 stallions with a median (range) age of 9.8 [2.0 d-27.7 yrs] years and a body mass of 540 [56–775] kg. This number of horses represent 76.7% of the horses that underwent general anesthesia during the study period, as some horses had to be excluded due to lack of photographs or behavioral assessment prior to general anesthesia. Breeds represented comprised warmbloods (170), ponies (70), draft horses (10), thoroughbreds (9), and horses from other breeds (26). Two hundred three horses (67.7%) underwent elective procedures and 97 (32.3%) were emergency procedures. In 37 (12.3%) of the 300 cases, general anesthesia was maintained by TIVA, whereas 263 horses (87.7%) received inhalational anesthesia. During 35 (94.6%) of the TIVA cases, only EtCO_2_ was measured compared to PaCO_2_, while arterial blood gas analysis was performed for each volatile anesthesia. Overall, the anesthetic duration (median, range) was 112 [10–448] minutes, inhalational anesthesia took 125 [16–448] minutes and TIVA 22 [10–72] minutes. The median (range) scores were 2 [1–5] for the ASA-PS-Equine, 13 [10–18] for the 10-RS, and 15 [11–22] for the CHARIOT (Table 1). Distribution of the horses into the individual categories of the 10-RS are presented in Table 2.

**Table 1:** Distribution of 300 Horses into the classes of a modified ASA system (ASA-PS-Equine), a 10-part rubic risk scale (10-RS), and a combination of both (CHARIOT) proposed by Hubbell et al. (7).

Score	Classification	Horses enrolled (n)
ASA-PS-Equine	1	24
	2	160
	3	67
	4	48
	5	1
10-RS	10	15
	11	40
	12	56
	13	59
	14	44
	15	44
	16	28
	17	10
	18	4
CHARIOT	11	5
	12	16
	13	35
	14	52
	15	48
	16	31
	17	25
	18	42
	19	22
	20	13
	21	10
	22	1

**Table 2:** Classification and risk assessment of 300 equine patients into the 10 categories of the 10-part rubric risk scale (10-RS) proposed by Hubbell et al. (7).

Variable	Category	Assigned risk	n
Age	< 3 month	2	6
	3 month to 15 years	1	214
	> 15 years	2	80
Weight	< 700 kg	1	294
	> 700 kg	2	6
Tractability	Easily handled	1	250
	Difficult to handle	2	50
Mobility	Normal	1	227
	Lame of mild ataxic	2	66
	Severely ataxic or recumbent	3	7
Pain Level	Controllable	1	216
	Uncontrollable	2	84
Abdominal profile	Flat bellied	1	124
	Round bellied	2	176
Procedure			1	131
		2	156
		3	13
Recumbency	Lateral	1	157
	Dorsal	2	143
Estimated anesthetic duration	< 90 min	1	131
	90 min to 3 h	2	150
	> 3 h	3	19
Recovery	Assisted recovery	1	300
	Free recovery	2	0

### 3.2 Intra-anesthetic complications

One or more intra-anesthetic complication occurred in 66.7% of the horses (Table 3). The CHARIOT was associated with a greater number of complications during general anesthesia than the ASA-PS-Equine or 10-RS alone (Table 4). The discriminant ability of the ASA-PS-Equine (AUC = 0.6093; 95% CI 0.5506– 0.6681), the 10-RS (AUC = 0.6701; 95% CI 0.6080–0.7323), and CHARIOT (AUC = 0.6697; 95% CI 0.6079–0.7315) related to the occurrence of intra-anesthetic complications was found to be low.

**Table 3:** Prevalence of intra-and post-anesthetic complications in 300 horses undergoing general anesthesia separated into maintenance with volatile or total intravenous anesthesia.

Variable	Number of observations	Inhalational anesthesia (*n* = 263)	Total intravenous anesthesia (*n* = 37)
**Intra-anesthetic complication**			
Arrhythmia	300	12	1
Bleeding	300	5	-
Intra. awake	300	112	9
Excitation	300	9	1
Hypercapnia	292	55	2
Hypotension	281	76	3
Hypothermia	99	15	1
Hypoxemia	292	46	-
Euthanasia	300	15	1
Anesthesia-associated mortality	300	-	-
Total		345	18
**Post-anesthetic complication**			
Epistaxis	284	5	-
Colic	282	16	1
Fractures	284	2	-
Myopathy	284	1	-
Neuropathy	284	11	1
Respiratory disease	282	13	1
Thrombophlebitis	282	6	-
Euthanasia	284	14	-
Anesthesia-associated mortality	284	2	-
Total		70	3

**Table 4:** Results of binominal logistic regression for the ASA-PS-Equine, 10-RS and CHARIOT for intra-anesthetic complications.

Variable	ASA-PS-Equine			10-RS			CHARIOT		
	*p*-value	Odds Ratio	95% CI	*p*-value	Odds Ratio	95% CI	*p*-value	Odds Ratio	95% CI
Arrhythmia	0.118	1.621	0.884–2.973	0.894	1.020	0.759–1.371	0.511	1.077	0.863–1.344
Bleeding	**0.013**	**4.426**	**1.363–14.378**	0.087	1.526	0.941–2.475	**0.029**	**1.565**	**1.046–2.341**
Intra. awake	0.294	1.153	0.884–1.504	**0.019**	**1.162**	**1.025–1.317**	**0.031**	**1.110**	**1.009–1.220**
Excitation	0.521	0.776	0.357–1.684	0.588	0.909	0.643–1.284	0.524	0.917	0.703–1.197
Hypercapnia	**< 0.001**	**1.850**	**1.330–2.574**	**< 0.001**	**1.462**	**1.238–1.728**	**< 0.001**	**1.348**	**1.188–1.530**
Hypotension	**0.001**	**1.665**	**1.230–2.252**	**< 0.001**	**1.327**	**1.145–1.538**	**< 0.001**	**1.253**	**1.121–1.400**
Hypothermia	0.464	0.775	0.392–1.533	0.414	1.135	0.838–1.538	0.707	1.408	0.822–1.335
Hypoxemia	**< 0.001**	**1.968**	**1.375–2.816**	**< 0.001**	**1.388**	**1.165–1.655**	**< 0.001**	**1.319**	**1.153–1.508**
Overall morbidity	**0.001**	**1.654**	**1.220–2.242**	**< 0.001**	**1.414**	**1.222–1.637**	**< 0.001**	**1.303**	**1.166–1.457**
Euthanasia	**< 0.001**	**5.314**	**2.550–11.074**	**< 0.001**	**1.717**	**1.275–2.312**	**< 0.001**	**1.742**	**1.345–2.256**

Statistically significant values are shown in bold letters.

### 3.3 Recovery phase

In total, 284 recoveries were analyzed. The number of attempts to stand (median, range) was 1 [1–10] and duration of recovery was 42 [10–165] minutes. Median (range) quality of recovery due to the modified scale of Young and Taylor (24) was 2 [1–5].There was a statistically significant association with all 3 scores and the number of attempts to stand (ASA-PS-Equine *p* = 0.0186; 10-RS *p* = 0.0077; CHARIOT *p* = 0.0490) and quality (ASA-PS-Equine *p* = 0.0038; 10-RS *p* < 0.001; CHARIOT *p* < 0.001) of the recovery phase. All 3 scores (*p* < 0.001 for all) showed a correlation with the duration of recovery, but low Spearman correlation coefficients (ρ) indicated only moderate strength of correlation (ASA-PS-Equine ρ = 0.3278, 10-RS ρ = 0.3552, CHARIOT ρ = 0.3729).

### 3.4 Post-anesthetic complications

The occurrence of fractures, myo-, neuropathies, epistaxis, and death/euthanasia after general anesthesia was evaluated in 284 horses. Due to limb fractures of 2 horses during recovery that required euthanasia, the complications “post-anesthetic colic,” “thrombophlebitis” and “respiratory disease” were studied in 282 horses, as only this number of horses survived long enough to express these pathologies. In total, 16.9% of the horses experienced post-anesthetic complications. All 3 scores were associated with sporadic pathologies following general anesthesia. The ASA-PS-Equine was associated with post-anesthetic respiratory disease (*p* = 0.0409) and death/euthanasia of a patient (*p* < 0.001). The occurrence of a thrombophlebitis (*p* = 0.038; *p* = 0.044), death/euthanasia (*p* < 0.001; *p* < 0.001), and overall morbidity (*p* = 0.008; *p* = 0.023) was associated with the 10-RS and CHARIOT. The discriminant ability of the ASA-PS-Equine (AUC = 0.5373; 95% CI 0.4586–0.6161), the 10-RS (AUC = 0.6194; 95% CI 0.5352–0.7037), and CHARIOT (AUC = 0.6062; 95% CI 0.5214–0.6911) related to the occurrence of post-anesthetic complications was low.

### 3.5 Mortality

**Figure 1:**
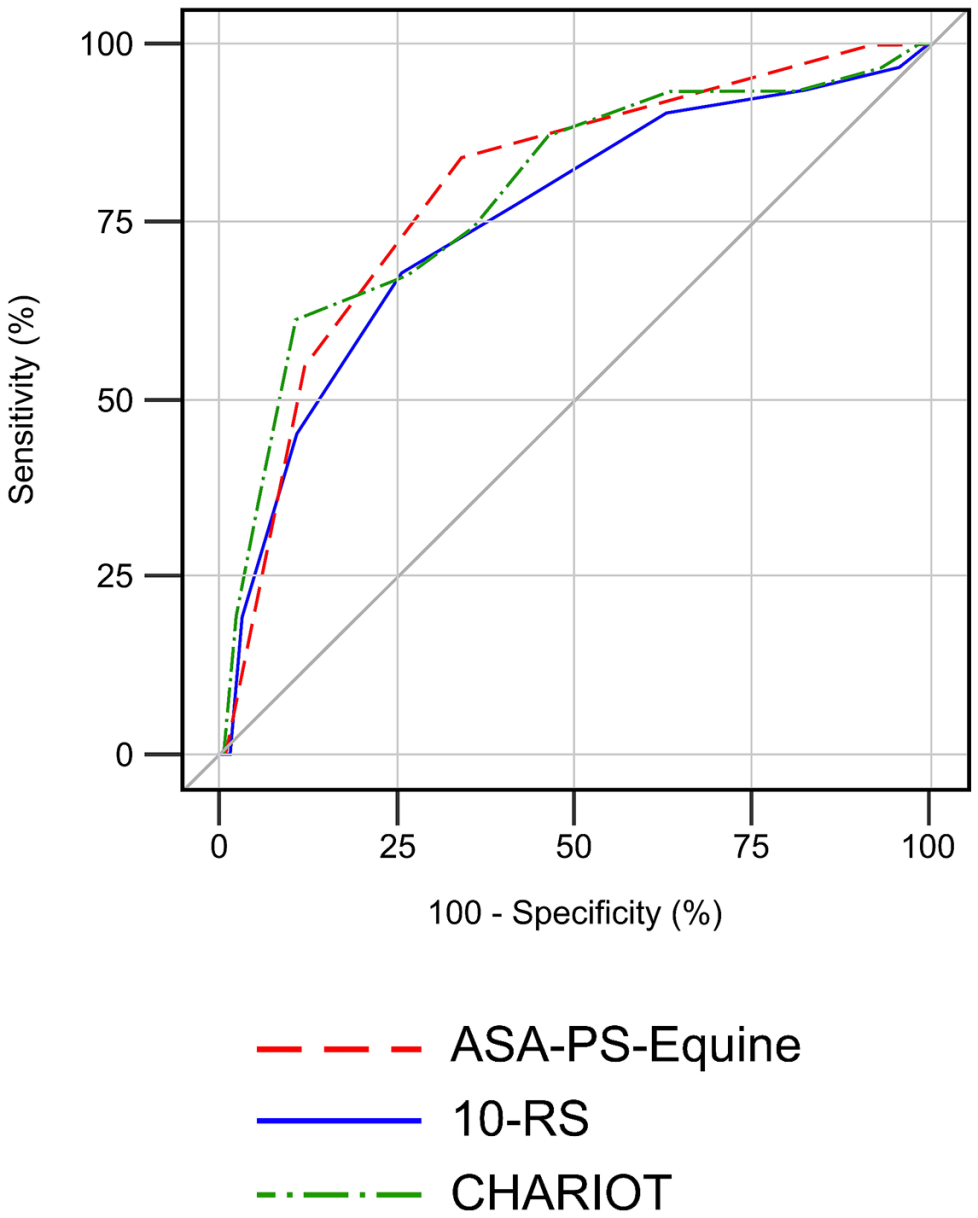
Receiver operating characteristics curve analysis comparing the ability of the ASA-PS-Equine (AUC = 0.7970; 95% CI 0.7199–0.8741), 10-RS (AUC = 0.7526; 95% CI 0.6558–0.8494), and CHARIOT (AUC = 0.7930; 95% CI 0.7004–0.8855) to predict the overall mortality rate of 300 horses over a period of 7 days following general anesthesia.

The overall mortality rate was 10.7% and anesthesia-associated mortality was 0.67%. Intra-operatively, 16 horses (5.33%) had to be euthanized due to inoperable lesions or poor prognosis found at surgery in combination with limited financial resources or the owner’s request. In the post-recovery period, a further 14 horses (4.93%) were euthanized due to recurrent colic symptoms (*n* = 6), severe systemic inflammatory response syndrome (*n* = 2), septic peritonitis (*n* = 2), evisceration (*n* = 1), corneal perforation (*n* = 1), idiopathic epilepsy (*n* = 1), and podotrochlosis (*n* = 1). In association with anesthesia, two patients died due to limb fractures (tibia fracture, fracture of the third metatarsal bone) during the recovery phase. Prognostic performance of the ASA-PS-Equine (AUC = 0.7970; 95% CI 0.7199–0.8741), 10-RS (AUC = 0.7526; 95% CI 0.6558–0.8494), and CHARIOT (AUC = 0.7930; 95% CI 0.7004–0.8855) was most accurate when the overall mortality rate was examined. In this context, the ASA-PS-Equine differentiated most precisely (Figure 1). The odds of peri-anesthetic death increased approximately 5.3-fold for each one-unit increase in ASA-PS-Equine grade for intra-anesthetic euthanasia and 2.7-fold for post-anesthetic euthanasia.

### 3.6 Validity and reliability

All 12 veterinarians (100%) completed the questionnaire including hypothetical patients. The median (range) age of the 10 female and 2 male participants was 27.5 [24–39] years. Median (range) clinical experience was 4 [1–14] years and all veterinarians surveyed were currently undergoing or had completed horse related specialization at the time of the survey. The majority of veterinarians (*n* = 8; 66.67%) rated the speed with which horses can be categorized into the CHARIOT as quick or very quick. Ten out of 12 (83.33%) participants ranked the ease of use as rather easy and half of them (*n* = 6; 50%) felt rather confident in its use. Eleven of 12 (91.67%) experts endorsed the use of the CHARIOT in clinical practice. When interrater reliability was considered, the ASA-PS-Equine (κ = 0.52) was superior to the 10-RS (κ = 0.39).

## 4 Discussion

The study demonstrates the overall usability of the CHARIOT in clinical practice. Both the CHARIOT and its individual parts, the ASA-PS-Equine and the 10-RS, provide useful information for the prediction of anesthesia-associated mortality in the studied equine patients, whereas the discriminatory power for the prediction of specific intra-and post-anesthetic pathologies is weak. The combined CHARIOT did not improve mortality risk prediction compared to the single components in the studied population.

In the investigated population of horses, an association between the ASA-PS-Equine, 10-RS, and the CHARIOT and various intra-anesthetic complications was shown. In addition, the CHARIOT was associated with a greater number of intra-anesthetic complications than the ASA-PS-Equine and 10-RS alone (Table 4). This may be due to the fact that the CHARIOT comprises patient-specific as well as procedure-specific and anesthesia-specific risk factors. While, to the knowledge of the authors, an association between the ASA status and peri-anesthetic morbidities has not yet been demonstrated in horses, it was shown that canine patients with higher ASA grades are prone to longer intensive care unit stays following general anesthesia, possibly indicating more severe peri-operative complications in these patients (27). Associations between several categories of the 10-RS or CHARIOT and peri-anesthetic complications are reported in the literature. Lower arterial blood pressure values have been described for horses in dorsal recumbency compared to lateral recumbency during elective procedures under inhalational anesthesia (28). Higher body weight and the type of procedure performed can predispose horses to hypoxemia during general anesthesia. In horses undergoing exploratory laparotomy for instance, the risk of developing hypoxemia is more than six times higher than in horses undergoing elective procedures (29).

All 3 scores were associated with the number of attempts to stand, the duration, and quality of the recovery phase. An association between higher ASA grades and reduced recovery quality has previously been demonstrated in horses (1, 30). Additionally, the influence of several factors contained in the 10-RS on the duration of recovery has already been documented. The horse’s temperament (31), anesthetic duration (1, 24, 32) and the type of procedure performed (32) may alter recovery time without assistance. The effects of assistance on the duration of recovery are diverging. While Arndt et al. (23) described shorter recovery times after elective procedures and Luoro et al. (33) better quality of recoveries after exploratory laparotomy, Rüegg et al. (34) were unable to demonstrate a reduction in recovery time after emergency abdominal surgery by providing assistance. Overall, the literature already indicates a possible association with recovery time for 4 out of 10 parameters of the 10-RS.

The association between the ASA grade and peri-operative mortality in dogs and cats (27, 35–37), rabbits (35), and horses (1, 38) is well documented. The same can be verified for the ASA-PS-Equine in the present study. While Dugdale et al. (1) reported, that the odds of peri-anesthetic death increased approximately 2.85-fold for each one unit increase in ASA grade, we found an almost doubling increase for intra-anesthetic euthanasia and a comparable increase for post-anesthetic euthanasia for the ASA-PS-Equine in our study population. However, due to differing definitions of mortality, data might not be directly comparable. The study by Dugdale et al. (1) does not indicate whether the calculation between the ASA grade and mortality risk also includes euthanized horses. If not, the high number of euthanized horses during general anesthesia in our study (*n* = 16, 5.3%) could be responsible for the deviating results.

Although the study documented a statistically significant association of the 10-RS and CHARIOT with intra-anesthetic or post-anesthetic euthanasia, the ASA-PS-Equine shows the greatest discriminatory power in this regard. It is possible that the overall condition of the horse most strongly influences the likelihood of euthanasia and that procedure-related risk factors are less relevant.

The inter-observer reliability of the ASA-PS-Equine was found to be moderate (κ = 0.52). Several studies in human medicine have documented κ-values of 0.2–0.4 for the agreement of ASA assessments in hypothetical patients (11, 15, 39). Similar results have been described in small animals by predominantly anesthesia specialists (12) or general practitioners (40). The improved agreement in the present study could be explained by the addition of exemplary equine-specific diseases to the original ASA classification. In humans, a web-based questionnaire study documented that both anesthesia-trained and nonanesthesia-trained clinicians were more likely to correctly classify the ASA if exemplary diseases had previously been assigned (41). However, the validation of the current ASA version from 2014, supplemented with example diseases, is also still pending in human medicine (26).

Apart from the equine-specific CHARIOT, only the LeiV-Risk-Index (39) is currently available as a multi-factorial index for the pre-anesthetic risk assessment in dogs. It is inter-observer reliability is superior to the CHARIOT with κ = 0.55 (42), but comparability is limited due to diverging categories, scoring system and number of hypothetical patients analyzed. A comparison of the κ-values for the 10-RS and ASA-PS-Equine shows that the reliability of the ASA-PS-Equine assessment is higher. The smaller extent of the ASA classification, its simplicity itself (40) or the simplification of the assignment to a class by the disease examples might have led to an improvement in performance.

The current study has some methodological limitations that should be considered. It is unclear how the experience of different anesthetists, varying anesthetic protocols, and possible insufficient documentation affected the prevalence of intra-anesthetic complications in particular. While level of training and clinical experience of anesthetists regularly do not affect the mortality risk in horses undergoing general anesthesia, there is evidence that cases treated by highly skilled anesthetists contain an increased risk (3). This is presumably due to the fact that anesthesia for the most difficult or risky operations is often performed by the best-qualified personnel (3). Several studies have already demonstrated that anesthetic mortality can be influenced by the use of different medications and anesthetic management (1, 3, 43). For example, premedication of adult horses with acepromazine and maintenance with ketamine-TIVA can reduce the risk of death in horses (1, 3, 4, 43), whereas the administration of different intravenous induction agents in combination with inhalational maintenance is not associated with outcome (3). The absence of premedication, or purely inhalational anesthesia, especially when halothane is used, increases the risk of death (3). Anesthetic maintenance can also have an effect on the development of intra-and post-anesthetic complications. While horses are particularly sensitive to the negative inotropic and vasodilatory effects of volatile anesthetics (44), hypotension rarely occurs during ketamine-TIVA (45, 46). Besides other predisposing factors, a reduction in muscle perfusion due to hypotension may increase the risk of developing neuromuscular pathologies during general anesthesia (24, 47, 48) and prolong recovery time (32). In addition, maintenance of anesthesia with ketamine-TIVA is predominantly used for shorter cases, and it is well known that the duration of anesthesia strongly influences the morbidity- and mortality rate of equine patients. While peri-operative mortality increases with anesthetic duration of 61 min, it is highest exceeding 241 min compared to the control group that underwent general anesthesia for less than 30 min (4). A low mortality rate of 0.24% within 7 days post-operatively was found in a monocentric study that mainly investigated elective procedures with anesthesia times of less than 1 h (49). This contrasts with anesthesia-related mortality rates of approximately 1% from studies conducted between 1984 and 2016 (1–5, 24, 50, 51). As the proposed version of the CHARIOT does not take the type of anesthesia or the medication used into account, these factors were also excluded when conducting the study. In human medicine, missing documentation is widespread and is a source of clinical data miscoding in 56% of cases (52), however, electronic documentation, as performed in the clinic for horses, minimizes the occurrence of documentation errors (53).

Additionally, the limited study population of 300 horses and the small number of horses in some of the categories might have negatively influenced the informative value of the study. Because of the lack of published data, a sample size calculation based on CHARIOT was not possible. Nevertheless, the evaluated population reflected the presented patients during the study period and the scope of the study allows a first, preliminary evaluation of the CHARIOT, which can be used as a baseline for larger, multicentric studies. Besides that, due to the clinic specific spread of patients, it was rarely possible to assign very high scores and no maximum scores for the CHARIOT, which may have led to less robust results. The high percentage of emergency procedures (*n* = 97, 32.3%) with exploratory laparotomies accounting for the majority of cases (*n* = 79, 26.3%), does not correspond to the distribution of procedures in multi-center studies like CEPEF 1 and 2 (3, 4) and the preliminary results of CEPEF 4 (6). Therefore, the transfer of the results to equine clinics with different surgical priorities and caseloads can be limited.

In conclusion, a better predictive power of the CHARIOT compared to its single parts could not be proven in this study. The CHARIOT, 10-RS and ASA-PS-Equine are associated with the peri-anesthetic mortality rate in horses and especially the CHARIOT may sensitize anesthetists in form of a checklist for high-risk patients. The addition of exemplary diseases to the ASA classification, which generates the ASA-PS-Equine, improved inter-observer reliability compared to traditional ASA assessment, as reported in the literature, and allowed for a more precise differentiation of the mortality risk of equine patients compared to the 10-RS and CHARIOT. A larger, multi-center study involving a more heterogeneous sample of horses would contribute to a more universally applicable evaluation of the CHARIOT.

## Data availability statement

The raw data supporting the conclusions of this article will be made available by the authors, without undue reservation.

## Ethics statement

The requirement of ethical approval was waived by Institutional Ethical committee of the University of Veterinary Medicine Hannover for the studies involving animals because the study was purely observational in nature and there was no intervention to any animal as a result of the study. The studies were conducted in accordance with the local legislation and institutional requirements. Written informed consent was obtained from the owners for the participation of their animals in this study.

## Author contributions

LB: Conceptualization, Data curation, Funding acquisition, Investigation, Methodology, Project administration, Resources, Validation, Visualization, Writing – original draft, Writing – review & editing. LW-V: Conceptualization, Formal analysis, Investigation, Methodology, Supervision, Validation, Writing – review & editing. KR: Conceptualization, Investigation, Software, Writing – review & editing, Methodology, Visualization. SK: Conceptualization, Formal analysis, Methodology, Supervision, Validation, Writing – review & editing.
