# Correction: Risk assessment in equine anesthesia: a first evaluation of the usability, utility and predictivity of the two-part CHARIOT

**DOI:** 10.3389/fvets.2025.1685121

**Published:** 2025-09-11

**Authors:** Lisa Brumund, Liza Wittenberg-Voges, Karl Rohn, Sabine B. R. Kästner

**Affiliations:** ^1^Clinic for Horses, University of Veterinary Medicine Hannover, Foundation, Hannover, Germany; ^2^Institute for Biometry, Epidemiology and Information Processing, University of Veterinary Medicine Hannover, Foundation, Hannover, Germany; ^3^Clinic for Small Animals, University of Veterinary Medicine Hannover, Foundation, Hannover, Germany

**Keywords:** horse, equine, anesthesia, mortality, morbidity, risk assessment, CHARIOT

In the published article, there was an error in Results, *Intra-anesthetic complications*. The sentence previously stated,

“The discriminant ability of the ASA-PS-Equine (AUC = 0.6093; 95% CI 0.5506–0.6681), the 10-RS (AUC = 0.6701; 95% CI 0.6080–0.7323), and CHARIOT (AUC = 0.6697; 95% CI 0.0315–0.6079) related to the occurrence of intra-anesthetic complications was found to be low.”

The corrected sentence appears below:

“The discriminant ability of the ASA-PS-Equine (AUC = 0.6093; 95% CI 0.5506–0.6681), the 10-RS (AUC = 0.6701; 95% CI 0.6080–0.7323), and CHARIOT (AUC = 0.6697; 95% CI 0.6079–0.7315) related to the occurrence of intra-anesthetic complications was found to be low.”

The original article has been updated.

